# Persistent organic pollutants in meat, liver, tallow and bone marrow from semi-domesticated reindeer (*Rangifer tarandus tarandus L*.) in Northern Norway

**DOI:** 10.1186/1751-0147-55-57

**Published:** 2013-08-13

**Authors:** Ammar Ali Hassan, Charlotta Rylander, Magritt Brustad, Torkjel M Sandanger

**Affiliations:** 1 Centre for Sami Health Research, Department of Community Medicine, Faculty of Health Sciences University of Tromsø, N-9037, Tromsø, Norway; 2 Department of Community Medicine, Faculty of Health Sciences University of Tromsø, N-9037, Tromsø, Norway

**Keywords:** Reindeer, POPs, PCBs, DDTs, OCPs, PBDEs, Arctic food, Sami, Norway

## Abstract

**Background:**

The aim of this project was to study 14 polychlorinated biphenyls (PCBs), 5 dichlorodiphenyl trichloroethans (DDTs), 12 organochlorine pesticides (OCPs) and 6 polybrominated diphenylethers (PBDEs) in meat, liver, tallow and bone marrow from semi-domesticated reindeer.

**Methods:**

Meat, liver, tallow, and bone marrow samples (n= 30) were collected from semi-domesticated reindeer in Northern Norway. Determination of the persistent organic pollutants (POPs) concentrations was done by using gas chromatography–mass spectrometry (GC-MS). Dependent sample t-test and Pearson’s correlation test were used in statistical analysis.

**Results:**

Concentrations of the persistent organic pollutants in the samples from semi-domesticated reindeer were generally low and slightly above the limit of detection (LOD). For PCBs and OCPs, ≥ 50% of the samples had concentrations above LOD. For the DDTs and PBDEs, the proportion of samples with concentrations above LOD varied between 3.7 and 45.5% depending on the sample type. Concentrations of PCB 99, 105, 138/163, 153 and 187 differed significantly between meat and liver, whereas concentrations of PCB 183 were significantly different between tallow and bone marrow. Furthermore, concentrations of hexachlorobenzene (HCB) were significantly different between meat and liver. Significant correlations were revealed in concentrations of 5 PCB congeners between the studied tissue types.

**Conclusion:**

Concentrations of the POPs revealed in this study were generally low.

## Background

Persistent organic pollutants (POPs) are a variety of environmental contaminants that are resistant to degradation, have the ability to bio-accumulate and are toxic to humans and the environment. POPs can be transported over long distances, especially by air currents, resulting in deposition and accumulation in areas far from the release source [1-4]. Most POPs are fat soluble (lipophilic), which is the reason why they are present in high concentration in lipid rich tissues. The content in meat with low fat content such as reindeer meat is therefore expected to be low [5]. POPs are slowly released from adipose tissues and into the bloodstream thereby having the potential to be harmful for human health [6].

Polychlorinated biphenyls (PCBs) have previously been used for a range of industrial purposes such as printer’s ink and electric motors and have been classified as POPs. Around 100 different PCB congeners have been detected in biological samples [7,8]. Organochlorine pesticides (OCPs) are a large group of compounds which have been used for various pest controls. They are an issue of concern due to their direct use in natural systems. The use of dichlorodiphenyl trichloroethans (DDTs) as pesticides has declined and is restricted to use in the campaign against mosquito born tropical diseases, e.g. malaria and dengue fever [9,10]. Polybrominated diphenylethers (PBDEs) are another group of compounds used in textiles and in various electric equipment, as flame retardant [11,12].

Traditional food (*e*.*g*., blubber, wild bird eggs, etc.) is the main source of human exposure to POPs among indigenous people in the Arctic and at the same time an excellent source of nutrients [13]. Despite the large numbers of published articles on environmental contaminants in the Arctic, relatively few studies have been carried out on reindeer. The main focus has been given to marine mammals since levels of POPs have been low in the terrestrial ecosystem due to low fat content [14-16]. Despite that, it is necessary to gain knowledge of current POP concentrations in terrestrial animals since several of them are important part of human food chain.

Lichens, the main winter feed of reindeer, absorb and accumulate environmental pollutants due to their long survival, tolerability and fairly uniformed morphology compared to other type of plants [13,17]. Hence, semi-domesticated reindeer are mainly exposed to pollutants through contaminated lichens [18]. Climate variability and global climate change influence the routes and mechanisms by which pollutants are delivered to the Arctic and may increase accumulation of POPs in lichens [13,19]. Plants in general are considered to be the major source of POPs into terrestrial food chains [20]. Even though, levels of some POPs such as PCBs and DDTs in the Arctic are decreasing due to regulations and efforts made, others such as PBDEs and fluorinated compounds have been increasing due to increased global production and use of these contaminants [21].

The main aims of the present study were to determine concentrations of POPs in semi-domesticated reindeer from Northern Norway and to explore differences in concentrations between meat and the other edible reindeer products (liver, tallow and bone marrow). We also aimed to study correlations of POPs between the different tissue types.

## Methods

### Sample collection

Muscle, liver, tallow and bone marrow samples were collected from semi-domesticated reindeer from Finnmark and Nordland counties in Northern Norway. The number of samples (n) varied between 30 and 19, due to factors such as inadequate sample quantity. The average age of the reindeer from which samples were collected from September 2004 to January 2005 was young (1.5 year). However, a limited number of calves (20%) and adult animals (10%) were chosen because of scarcity of slaughtered young animals. Age of the reindeer was obtained directly from the tags attached to animals’ carcasses when they passed the weighing post in the slaughterhouses.

Meat samples were collected from muscles of the dorsal neck. Liver samples were collected from the main lobe, renal tallow was collected from the part attached to the back and bone marrow was collected from the fore (metacarpus) and hind (metatarsus) limb bones. All samples were collected directly after slaughter/ dressing process and immediately after carcass weighing in pre-marked plastic bags prior to further division in dedicated glass containers. Each glass was covered with aluminium foil, and then labelled with sample type, carcass number, district name/ number and date of sample collection. The samples were kept cool in a cooling box (approximately 4°C) immediately after collection and division, and then moved the same day to a – 20°C freezer until they were freighted frozen to the laboratory for analysis. Precautionary measures were taken during sample collection to avoid possible environmental contamination of the samples.

### Analytical procedures

POP analysis was performed at the NILU (Norwegian Institute for Air Research) Laboratory, Tromsø, Norway; accredited for the methods used in the analyses according to NS-EN ISO/IEC 17025, No. TEST008.

Tissue samples (meat, liver, tallow and bone marrow) of 2–5 g each were separately homogenised by addition of pre-treated sodium sulphate Na_2_SO_4_ (600°C, 8 h) in a ratio of 1:20. 20 μl ^13^C-isotope labelled internal standards were added to each sample. The homogenised mixture was extracted three times using cold column extraction with 50 ml cyclohexane: acetone (3:1; v/v). The extract (150 ml) was then concentrated to 0.5 ml and collected in a 4 ml vial [22]. The amount of extractable lipid was determined gravimetrically.

The extracts were cleaned-up using gel permeation chromatographic (GPC) and pre-packed florosil columns prior to analyses on a gas chromatography–mass spectrometry (GC-MS) instrument. This procedure has been presented in detail elsewhere [22]. The quality of the methods used was regularly verified and samples were quality assured during analysis using both blank and standard reference material (SRM) samples between sets of 5 biological samples each.

### Statistical analysis

Laboratory results for persistent organic pollutants (POPs) below the limit of detection (LOD) were given a numeric value at half the detection limit (LOD/2) according to statistical practice of non-detects data [23]. Data with concentrations below the LOD (< LOD) in ≥ 50% of the total samples were not included in the statistical analyses and were presented as range (<LOD –maximum detected concentration). STATA/SE 12.0 for Windows (STATA Corp. College Station, TX) was used for statistical analyses. Data were positively skewed (skewed to the right), hence log transformation was performed. Dependant sample paired t-test was used to test for differences in POP concentrations between meat and liver, and tallow and bone marrow. Pearson correlation test was used to test for significant correlations. The level of significance was set at (p< 0.05) for all statistical analyses. Results on POPs are presented as percentage of detected pollutants, arithmetic mean (AM) ± SD, geometric mean (GM) and range (Min. – Max.). POP concentrations are presented in ng/g wet weight (ww) rather than lipid weight (lw) as it is easier to relate to wet weight for consumers in addition to the fact that reindeer meat contains little fat (2%).

## Results and discussion

Mean lipid percentages in meat, liver, tallow and bone marrow measured from this study were 2±0.84%, 5.6±0.88%, 78.9±6.84% and 71.7±15.99%, respectively. Meat, liver, tallow and bone marrow from semi-domesticated reindeer in Norway contained low levels of POPs that were in most cases similar to or slightly above the LOD. Significant correlations (0.01< p <0.05) were revealed in concentrations of 5 PCB congeners between the studied tissue types. Many values below detection limits in this study make it difficult to perform statistical analyses on all the studied compounds and have hence limited our ability for a broad comparison.

### Polychlorinated biphenyls (PCBs)

PCB concentrations in the different tissues are provided in Table 1. Concentrations of PCB 99, 105, 138/163, 153 and 187 were significantly lower in meat (GM= 0.03, 0.03, 0.06, 0.06 and 0.01 ng/g ww, respectively) than in liver (GM= 0.05, 0.06, 0.16, 0.24 and 0.05 ng/g ww, respectively). This could largely be explained by the fact that meat contained less fat than liver. Additionally, our samples were collected from calves, young and older animals with young animals representing the majority. Evidence of age-related variation in concentration of PCBs in meat and liver from Finnish reindeer has previously been reported [24-27]. The meat and liver of Finnish reindeer calves contained higher PCB concentrations than adult animals which have been explained by transfer of contaminants in reindeer dam to calves, mainly through lactation and to a lesser degree via placental routes. Due to the limited number of calves and adult animals combined by many levels below the limit of detection in the present study, we could not investigate the effect of age on concentrations of PCBs and the rest of POPs. Concentrations of PCB 183 were significantly higher in tallow than in bone marrow (GM= 0.12 and 0.08 ng/g ww, respectively). The correlations of PCB concentrations between the different tissues are presented in Table 2.

**Table 1:** Concentrations (ng/g ww) of polychlorinated biphenyls (PCBs) in meat, liver, tallow and bone marrow from reindeer

**Compound**	**Meat (% fat; mean: 2**, **range: 0**.**8 – 3.8)**						**p-value**	**Liver (% fat; mean: 5.6, range: 4.4 – 8.6)**					
	**n**	**%>LOD**	**GM**	**AM±SD**	**Min. - Max.**	**LOD**		**n**	**%>LOD**	**GM**	**AM±SD**	**Min. - Max.**	**LOD**
PCB 28	21	9.5			<0.12 – 0.12	0.12		28	0			<0.11	0.11
PCB52	24	33.3			0.04 – 0.16	0.04		20	0			<0.06	0.06
PCB 99	28	53.7	0.03	0.06±0.09	0.02 – 0.52	0.02	*	28	89.3	0.05	0.06±0.03	<0.02 – 0.14	0.02
PCB101	29	44.8			<0.3 – 0.64	0.03		26	15.4			<0.06 - 0.07	0.06
PCB105	29	65.5	0.03	0.06±0.13	0.01 – 0.69	0.01	**	29	100	0.06	0.07±0.02	0.02 – 0.12	0.02
PCB 118	22	77.3	0.08	0.18±0.29	<0.03 – 1.41	0.03	n.s	27	100	0.13	0.13±0.06	<0.03 – 0.23	0.03
PCB138/163	29	51.7	0.06	0.11±0.17	0.06 – 0.58	0.04	**	25	100	0.16	0.18±0.07	0.07 – 0.40	0.06
PCB153	29	51.7	0.06	0.08±0.09	<0.04 – 0.48	0.04	**	29	100	0.24	0.27±0.15	0.10 – 0.71	0.10
PCB 156	27	51.9	0.01	0.02±0.02	<0.01 – 0.09	0.01		20	40			<0.01 – 0.03	0.01
PCB 170	25	24			<0.02 – 0.15	0.02		27	48.2			< 0.02 – 0.12	0.02
PCB 180	23	39.1			< 0.03 – 0.11	0.03		29	65.5	0.07	0.08±0.05	<0.02 – 0.22	0.02
PCB 183	23	13			<0.01 – 0.03	0.01		27	55.6	0.02	0.03±0.02	<0.01 – 0.08	0.01
PCB 187	26	53.9	0.01	0.02±0.01	< 0.01 – 0.06	0.01	**	29	75.9	0.05	0.05±0.03	<0.01 – 0.12	0.01
PCB 194	24	4.2			<0.04 – 0.07	0.04		16	0			<0.03	0.03
	Tallow (% fat; mean: 78.9, range: 72.6 – 86.1)							Bone Marrow (% fat; mean: 71.7, range: 58.9 – 89.6)					
PCB 28	27	33.3			<0.20 – 1.28	0.20		21	61.9	0.17	0.24±0.19	< 0.11 – 0.61	0.11
PCB52	21	9.5			<0.20 – 1.37	0.20		19	5.3			<0.11 – 0.13	0.11
PCB 99	28	82.1	0.46	0.59±0.46	< 0.32 – 2.13	0.32	n.s	21	90.5	0.36	0.47±0.31	< 0.03 – 1.32	0.03
PCB101	28	17.9			<0.15 – 2.60	0.15		21	57.1	0.10	0.12±0.06	< 0.08 – 0.24	0.08
PCB105	24	100	0.60	0.67±0.29	0.16 – 1.24	0.01	n.s	18	100	0.49	0.58±0.29	0.07 – 1.07	0.07
PCB 118	23	100	1.67	1.82±0.81	0.87 – 3.51	0.13	n.s	22	100	1.28	1.48±0.66	0.40 – 2.78	0.07
PCB138/163	24	95.8	1.15	1.43±0.96	< 0.24 – 3.65	0.24	n.s	21	100	1.38	1.69±0.99	0.13 – 4.71	0.13
PCB153	28	100	2.29	2.62±1.25	0.68 – 5.21	0.61	n.s	23	100	2.17	2.49±1.07	0.28 – 4.50	0.11
PCB 156	22	63.6	0.22	0.24±0.11	< 0.06 – 0.50	0.06	n.s	22	59.1	0.18	0.22±0.14	<0.03 – 0.48	0.03
PCB 170	25	88	0.69	0.74±0.25	< 0.10 – 1.24	0.10	n.s	23	100	0.57	0.66±0.31	0.12 – 1.47	0.06
PCB 180	26	100	1.16	1.33±0.71	0.26 – 3.42	0.12	n.s	23	100	0.93	1.11±0.58	0.14 – 2.57	0.06
PCB 183	26	100	0.12	0.15±0.10	0.04 - 0.48	0.04	**	23	60.9	0.08	0.09±0.07	<0.02 – 0.35	0.02
PCB 187	28	53.6	0.22	0.25±0.15	< 0.07 – 0.62	0.07	n.s	22	86.4	0.18	0.19±0.08	< 0.04 – 0.31	0.04
PCB 194	27	33			< 0.20 – 0.42	0.20		21	52.4	0.21	0.23±0.09	< 0.11 – 0.53	0.11

**Legend**: n: Total number of samples, *LOD* Limit of detection, % >LOD: Percentage of samples with concentrations above the LOD,

Min. – Max.: Minimum and maximum concentrations. Levels of statistical significance; *ns* Not significant, * = 0.01< p <0.05, ** = p< 0.01.

**Note**: Arithmetic (AM) and geometric (GM) means were not calculated in cases where percentage of samples over LOD were <50%.

**Table 2:** **Polychlorinated biphenyls** (**PCBs**) **correlations between meat**, **liver**, **tallow and bone marrow from reindeer**

**PCB congener**	Correlation coefficient (p-value) n.s,*, **					
	**Meat**-**liver**	**Meat**-**tallow**	**Meat**-**bone marrow**	**Liver**–**tallow**	**Liver**–**bone marrow**	**Tallow**–**bone marrow**
PCB 99	0.69**	0.34^n.s^	0.16 ^n.s^	0.54**	0.37 ^n.s^	0.45
PCB 105	0.50**	0.60**	0.38 ^n.s^	0.82**	0.61**	0.68**
PCB 138	0.55**	0.47*	0.22 ^n.s^	0.63**	0.63**	0.57*
PCB 153	0.69**	0.69**	0.30 ^n.s^	0.86**	0.41 ^n.s^	0.41 ^n.s^
PCB 156	--	-0.19 ^n.s^	-0.54*	--	--	0.32 ^n.s^

**Legend**: (−−)= The correlation was not calculated in cases where at least one of the correlation components had less than 50% of samples with concentrations above the limit of detection (LOD). Levels of statistical significance: n.s = not significant, *= 0.01< p <0.05, **= p< 0.01.

Concentrations of PCBs 105, 118, 156 of 0.27, 0.69, 0.08 and 0.29, 0.57, 0.05 ng/g fat have previously been measured in meat and liver, respectively, from northern Finnish reindeer [28]. The same study mentioned above has reported PCBs 105, 118 and 156 concentrations of 0.27, 0.86 and 0.09 ng/g fat in meat from moose (*Alces alces*) originated from similar area as for reindeer [28]. Based on the mean fat percentages in meat and liver from the Finnish and this study, we could infer that concentration of PCBs 105, 118 and 156 were somewhat higher in the present study.

### Organochlorine pesticides (OCPs)

Concentrations and detection frequency of the measured OCPs are presented in Table 3.

**Table 3:** Concentrations (ng/g ww) of organochlorine pesticides (OCPs) in meat, liver, tallow and bone marrow from reindeer

**Compound**	**Meat (% fat; mean: 2, range: 0.8 – 3.8)**						**p**-**value**	**Liver (% fat; mean: 5.6, range: 4.4 – 8.6)**					
	**n**	**%>LOD**	**GM**	**AM±SD**	**Min. - Max.**	**LOD**		**n**	**%>LOD**	**GM**	**AM±SD**	**Min. - Max.**	**LOD**
α-HCH	25	0			<0.03	0.03		27	81.5	0.06	0.08±0.05	<0.03 – 0.19	0.03
β-HCH	26	7.7			<0.10 – 0.14	0.10		27	85.2	0.42	0.51±0.26	<0.15 – 1.31	0.15
γ-HCH	26	0			<0.05	0.05		27	3.7			<0.05 – 0.25	0.05
HCB	25	100	0.56	0.62 ±0.27	0.31 – 1.14	0.09	*	27	92.6	1.89	2.56±1.28	<0.11 – 4.55	0.11
Heptachlor	26	19.2			<0.08 – 0.46	0.08		27	0			<0.07	0.07
TOX-50	26	7.7			<0.01 – 0.07	0.01		27	0			<0.02	0.02
Mirex	26	0			<0.02	0.02		27	55.6	0.10	0.11±0.06	<0.05 – 0.41	0.05
	Tallow (% fat; mean: 78.9, range: 72.6 – 86.1)							Bone Marrow (% fat; mean: 71.7, range: 58.9 – 89.6)					
α-HCH	23	78.3	0.36	0.43±0.20	< 0.19 – 0.69	0.19	n.s	17	88.2	0.66	0.85±0.38	< 0.14 – 1.31	0.14
β-HCH	23	0			<0.97	0.97		17	58.8	0.51	0.56±0.23	< 0.34 – 0.98	0.34
γ-HCH	23	0			<0.35	0.35		17	0			<0.19	0.19
HCB	23	100	35.82	37.83±11. 72	16.59 – 53.25	0.66	n.s	17	100	41.89	43.34±11.64	26.03 – 63.01	0.42
Heptachlor	23	30.4			<0.49 – 1.33	0.49		17	29.4			<0.26 – 1.05	0.26
t-NC	23	0			<0.23	0.23		17	5.9			<0.13 – 0.15	0.13

**Legend**: n: Total number of samples, *LOD* Limit of detection, % >LOD: Percentage of samples with concentrations above the LOD, Min. – Max.: Minimum and maximum concentrations. Levels of statistical significance; n.s: Not significant, *: p< 0.05. **Note** (compounds not included in the table)**:** Concentrations of the congeners t-CD, c-CD, c-NC and TOX-26 were below the limit of detection (< LOD) in all samples from meat, liver, tallow and bone marrow, whereas Mirex was only detected in samples from liver. Heptachlor was not detected in samples from meat and liver. Arithmetic (AM) and geometric (GM) means were not calculated in cases where percentage of samples over LOD were <50%.

HCB had the highest detection percentages with 100% in meat, tallow and bone marrow, and 92.6% in liver. The highest HCB concentrations were detected in bone marrow and tallow (GM= 41.89 and 35.82 ng/g ww, respectively). Furthermore, meat had considerably lower concentrations of HCB than in liver (GM= 0.56 and 1.89 ng/g ww, respectively, p< 0.05).

The measured HCB concentration of 35.82 ng/g ww in this study was lower than that previously measured in Canadian caribou fat (55 ng/g ww) and considerably higher than that of 12 ng/g ww measured in German roe deer fat [14,29]. The measured HCB in liver from this study (1.89 ng/g ww) was considerably lower than that of 10 ng/g ww measured in German roe deer liver [30]. Mean concentrations of HCB in caribou fat collected from different northwest territories in Canada ranged from 32.93 to 129.41 ng/g lipid [31]. Concentrations of α- Hexachlorocyclohexane (α-HCH) in liver, tallow and bone marrow were 0.06, 0.36 and 0.66 ng/g ww, respectively, whereas β-HCH concentrations were 0.42 and 0.51 ng/g ww in liver and bone marrow, respectively. Concentrations of total HCHs (α-, β-, γ-) of 0.48 and 0.36 ng/g ww in liver and tallow, respectively, detected from this study were considerably lower than those of 11.0 and 23.7 ng/g ww formerly measured in German roe deer liver and Canadian caribou fat, respectively [14,30]. Mirex was only detected in liver sample with a geometric mean concentration (0.10 ng/g ww) slightly above the LOD.

No significant correlations were observed for pesticide concentrations (with ≥ 50% samples above the LOD) within the same tissue (inter-correlation) and between the different tissues (intra-correlation).

### Dichlorodiphenyl trichloroethans (DDTs)

Percentage of samples with DDT concentrations above the LODs ranged from 5.3 to 45.5% depending on the compound. Hence, concentrations of DDTs were presented as minimum and maximum levels, Table 4. Further statistical analysis was not possible according to the criteria we have set for such analysis (concentrations detection above the LOD in ≥ 50% of the total samples). The DDT compounds; *o*,*p*´ -DDE and *o*,*p*´ -DDD concentrations were below the limit of detection (< LOD) in all the four studied samples.

**Table 4:** **Concentrations** (**ng**/**g ww**) **of dichlorodiphenyl trichloroethans** (**DDTs**) **in meat**, **liver**, **tallow and bone marrow from reindeer**

**Compound**	**Meat (% fat; mean: 2, range: 0.8 – 3.8)**					**Liver (% fat; mean: 5.6, range: 4**.**4 – 8.6v**				
	**N**	**n**	**% > LOD**	**Min. – Max.**	**LOD**	**N**	**n**	**% > LOD**	**Min. – Max**.	**LOD**
*p*,*p*´-DDT	27	2	7.4	0.03 – 0.13	0.02	27	0	0		0.07
*o*,*p*´-DDT/*p*,*P*-DDD	23	0	0		0.04	26	5	19.2	0.02 – 0.10	0.02
*p*,*p*´-DDE	23	3	13	0.10 – 0.89	0.08	23	2	8.7	0.11 – 0.13	0.08
	Tallow (% fat; mean: 78.9, range: 72.6 – 86.1)					Bone Marrow (% fat; mean: 71.7, range: 58.9 – 89.6)				
*p*,*p*´-DDT	25	0	0		0.40	19	1	5.3	<0.06 – 0.09	0.06
*o*,*p*´-DDT/ *p*,*p* -DDD	30	0	0		0.25	20	3	15	0.06 – 0.10	0.06
*p*,*p*´-DDE	24	8	33.3	0.49 – 3.01	0.48	22	10	45.5	0.34 – 0.68	0.26

**Legend**: N = total number of samples. n = number of samples in which PBDEs were detected. *LOD* Limit of detection. % >LOD = Percentage of samples with concentrations above the LOD. Min. – Max. = Minimum and maximum concentrations. **Note**: *o*,*p*´-DDE and *o*,*p*´-DDD concentrations were below the limit of detection (< LOD) in all samples from meat (N = 24), liver (N = 30/ 26), tallow (N = 30/ 25) and bone marrow (N = 23/ 29). The LODs for *o*,*p*´-DDE and *o*,*p*´-DDD were 0.26/ 0.19 for meat, liver (0.24/ 0.22), tallow (0.75/ 1.3) and bone marrow (0.41/ 0.72) ng/g ww, respectively.

*p*,*p*´ -DDE concentrations of 0.84 and 23 ng/g ww have previously been reported in fat of Canadian caribou and German roe deer [14,29]. Furthermore, concentrations of DDTs in liver from roe deer and fat in caribou of 21.0 and 2.0 ng/g ww, respectively, have formerly been reported [14,30].

### Polybrominated diphenylethers (PBDEs)

Five out of the six PBDE congeners were found with detectable concentrations depending on the sample type with detection percentages ranging from 3.7 to 36%. Similarly as for DDTs (low detection percentage), concentrations of PBDEs were presented as minimum and maximum levels, Table 5. PBDE 138 concentrations were below the limit of detection (< LOD) in all the four sample types.

**Table 5:** **Concentrations** (**ng**/**g ww**) **of polybrominated diphenylethers** (**PBDEs**) **in meat**, **liver**, **tallow and bone marrow from reindeer**

**Compound**	**Meat (% fat; mean: 2, range: 0.8–3.8)**					**Liver (% fat; mean: 5.6, range: 4.4–8.6)**				
	**N**	**n**	**% > LOD**	**Min.–Max**.	**LOD**	**N**	**n**	**% > LOD**	**Min.–Max.**	**LOD**
PBDE 47	26	9	34.6	0.03 – 1.05	0.02	29	7	24.1	0.04 – 0.07	0.03
PBDE 99	25	4	16	0.23 – 1.93	0.05	28	2	7.1	0.03 – 0.10	0.02
PBDE 100	26	1	3.9	<0.10 – 0.29	0.10	30	0	0		0.10
PBDE 153	27	1	3.7	<0.04 – 0.19	0.04	30	0	0		0.04
PBDE 154	27	1	3.7	<0.04 – 0.13	0.04	30	0	0		0.02
	Tallow (% fat; mean: 78.9, range: 72.6–86.1)					Bone Marrow (% fat; mean: 71.7, range: 58.9–89.6)				
PBDE 47	25	9	36	0.21 – 4.15	0.18	20	1	5	<0.15 – 0.27	0.15
PBDE 99	25	7	28	0.21 – 7.22	0.18	20	1	5	<0.15 – 0.27	0.15
PBDE 100	24	1	4.2	<0.57 – 1.54	0.57	20	0	0		0.31
PBDE 153	27	2	7.4	0.68 – 0.87	0.20	22	3	13.6	0.21 – 0.38	0.10
PBDE 154	27	2	7.4	0.39 – 0.64	0.14	22	0	0		0.08

**Legend**: N = total number of samples. n = number of samples in which PBDEs were detected. *LOD* Limit of detection. % >LOD = Percentage of samples with concentrations above the LOD. Min. – Max. = Minimum and maximum concentrations. **Note**: PBDE 138 concentrations were below the limit of detection (< LOD) in all samples from meat (N = 27), liver (N = 30), tallow (N = 27) and bone marrow (N = 22). The LODs for PBDE 138 were 0.02 for meat and liver, and 0.13, 0.07 ng/g ww for tallow and bone marrow, respectively.

Data on PBDEs from reindeer are scarce. PBDE concentrations measured in meat, liver, milk and brown adipose tissue samples from Finnish reindeer exhibited higher concentrations in adult animals compared to calves [24]. Additionally, the liver from reindeer in the Finnish study represented the tissue with the clearest difference displaying the highest concentrations among the above mentioned tissues. We could not compare the PBDE results from the present study to the Finnish study as different congeners were measured and the Finnish study only presented ∑PBDEs. The above mentioned study documented also the effective placental transfer of PBDEs in reindeer. Concentrations of PBDEs in Arctic herbivorous terrestrial animals (*e*.*g*., reindeer, moose) have been reported to be lower than carnivorous ones such as birds of prey (e.g., peregrine falcon) [16,32,33]. In liver samples from northern Norwegian moose, PBDE-47 and 99 were the major detected congeners with median concentrations of 0.24 and 0.26 ng/g lw, respectively [34].

Concentrations of POPs in reindeer detected in this study were generally low and negligible compared to those previously reported from animals regarded as main source of human exposure to POPs such as Arctic birds, fish and marine mammals [15,35]. The low detection percentages (> 50% of samples were below the LOD) revealed in most of the POPs in the present study did not allow for a broad comparison and discussion.

## Conclusions

The majority of concentrations for the analysed POP compounds in this study were below the LOD. Concentrations measured for the POPs in this study were generally low. Further research, which includes more animals from several different grazing districts, is needed to take into consideration factors that we could not investigate in this study (*e*.*g*., geographical variations) and inclusion of other persistent organic pollutants (*e*.*g*., dioxin, perfluorinated compounds). The elevated dioxin levels previously measured in a few reindeers from the Norwegian-Russian border need to be followed up [36].

## Abbreviations

GM: Geometric mean; AM: Arithmetic mean; GPC: Gel permeation chromatography; LOD: Limit of detection; POPs: Persistent organic pollutants; PCBs: Polychlorinated biphenyls; DL-PCBs: Dioxin-like PCBs; DDTs: Dichlorodiphenyl trichloroethans; DDD: Dichlorodiphenyl dichloroethane; DDE: Dichlorodiphenyl dichloroethylene; OCPs: Organochlorine pesticides; PBDEs: Polybrominated diphenylethers; HCB: Hexachlorobenzene; HCH: Hexachlorocyclohexane.

## Competing interests

The authors declare no competing interests.

## Authors’ contributions

Acquisition of funding, study design and general supervision of the research group: MB, TMS. Sample collection and chemical analysis: CR. Statistical analysis, interpretation of data and manuscript writing: AAH. All authors have critically reviewed and approved the final manuscript.
